# Purified complement C3b triggers phagocytosis and activation of human neutrophils via complement receptor 1

**DOI:** 10.1038/s41598-022-27279-4

**Published:** 2023-01-06

**Authors:** Elena Boero, Ronald D. Gorham, Emmet A. Francis, Jonathan Brand, Lay Heng Teng, Dennis J. Doorduijn, Maartje Ruyken, Remy M. Muts, Christian Lehmann, Admar Verschoor, Kok P. M. van Kessel, Volkmar Heinrich, Suzan H. M. Rooijakkers

**Affiliations:** 1 Department of Medical Microbiology, University Medical Center Utrecht, Utrecht University, Heidelberglaan 100, 3584 CX Utrecht, The Netherlands; 2 GSK, 53100 Siena, Italy; 3 Sanofi, Waltham, MA 02451 USA; 4 Department of Biomedical Engineering, University of California Davis, Davis, CA 95616 USA; 5 Laboratory of Dendritic Cell Biology, Department of Dermatology, University Hospital of Erlangen, Friedrich-Alexander-Universität (FAU) Erlangen-Nürnberg, 91052 Erlangen, Germany; 6 Department of Otorhinolaryngology, Technische Universität München and Klinikum Rechts der Isar, 81675 Munich, Germany

**Keywords:** Biological techniques, Cell biology, Immunology, Microbiology

## Abstract

The complement system provides vital immune protection against infectious agents by labeling them with complement fragments that enhance phagocytosis by immune cells. Many details of complement-mediated phagocytosis remain elusive, partly because it is difficult to study the role of individual complement proteins on target surfaces. Here, we employ serum-free methods to couple purified complement C3b onto *E. coli* bacteria and beads and then expose human neutrophils to these C3b-coated targets. We examine the neutrophil response using a combination of flow cytometry, confocal microscopy, luminometry, single-live-cell/single-target manipulation, and dynamic analysis of neutrophil spreading on opsonin-coated surfaces. We show that purified C3b can potently trigger phagocytosis and killing of bacterial cells via Complement receptor 1. Comparison of neutrophil phagocytosis of C3b- versus antibody-coated beads with single-bead/single-target analysis exposes a similar cell morphology during engulfment. However, bulk phagocytosis assays of C3b-beads combined with DNA-based quenching reveal that these are poorly internalized compared to their IgG1 counterparts. Similarly, neutrophils spread slower on C3b-coated compared to IgG-coated surfaces. These observations support the requirement of multiple stimulations for efficient C3b-mediated uptake. Together, our results establish the existence of a direct pathway of phagocytic uptake of C3b-coated targets and present methodologies to study this process.

## Introduction

The complement system is an essential part of the innate immune system that provides the first line of defense against infections. Undesired complement activation on human cells is associated with many medical conditions, including severe diseases such as cancer, Alzheimer’s disease, multiple sclerosis^1^.

The phagocytic uptake of microbes is enabled or greatly enhanced by opsonization, i.e. the tagging of microbial surfaces with plasma-derived host proteins^2^. Generally, the most important and abundant plasma proteins mediating phagocytosis are antibodies and activation products of complement C3. Whereas the production of specific IgGs requires repeated exposure to an infectious agent, complement opsonins are generated through innate recognition and thus can induce phagocytosis even before antibodies are produced. Once bound to a pathogen surface, IgGs and C3-derived protein fragments engage Fcγ-receptors (FcγRs) and complement receptors (CRs) of immune cells, respectively^3^. Advanced imaging methods recently have illuminated detailed functional and mechanical aspects of FcγR-dependent^4–6^ and CR3-dependent phagocytosis^3,7^. However, similarly detailed insights into uptake mechanisms mediated by complement receptors other than CR3 are lacking.

Complement proteins originally circulate in the bloodstream as inactive precursors that are rapidly activated upon contact with a foreign surface. All three major complement-activation pathways converge at the formation of C3 convertases that cleave C3 molecules to C3b (S1). The covalent attachment of C3b to the surface of a target cell is mediated by a highly reactive thioester domain (TED) that is hidden within C3, but becomes exposed upon cleavage to C3b^8^. Depending on environmental conditions such as the composition of the target surface and/or the presence of other immune cells, C3b can then be converted into iC3b and/or C3dg via proteolytic processing of immobilized C3b by host regulatory proteins (S1).

The receptor most often associated with phagocytosis of complement-opsonized targets is complement receptor 3 (CR3; also known as CD11b/CD18, Mac-1, or integrin α_M_β_2_), a heterodimeric cell surface receptor that is a member of the β_2_ integrin family^9–11^. The primary ligand of CR3 is iC3b, with which interacts with high affinity (*K*_*d*_ ∼ 10^−7^ to 10^−6^ M). On the contrary, CR3 interacts very weakly with C3b^12^. In fact, the crystallographic structure of the heterodimeric iC3b–CR3α reveals that the CR3 binding surfaces on iC3b are only available following extensive rearrangements upon proteolysis^13–15^. The main receptor of C3b on phagocytes is CR1 (CD35), a type I transmembrane glycoprotein consisting of 30 complement control protein repeats^1,3,16–24^. CR1 is expressed on many cell types and has various functions in immunity. It is best-known for its role as an immune adherence receptor on erythrocytes, capturing and transporting immune complexes to the liver^25,26^. Furthermore, CR1 can act as a cofactor of the Factor I-dependent cleavage of C3b into iC3b^14,23^. Some reports have associated CR1 with phagocytosis by neutrophils, but additional stimuli such as IgG or fibronectin may have been involved or were required to induce C3b-mediated phagocytosis in these studies^9,24,27–29^.

In this study, we combine immunobiological assays with modern surface biochemistry and single-live-cell manipulation to investigate C3b-mediated phagocytosis by human neutrophils. Instead of using target particles that were prepared by incubation in host plasma or serum^16,19,21,30,31^, we describe and employ model systems specifically designed to assess the functional role of purified C3b in neutrophil phagocytosis in the absence of other serum components.

Our data reveal previously overlooked roles of C3b in phagocytosis, demonstrating that purified C3b drives bacterial uptake and killing via CR1 in a serum-free environment. Furthermore, we show that the uptake of C3b-coated particles proceeds in a manner that is morphologically similar to the phagocytosis of IgG-coated beads of comparable size, with the formation of a phagocytic cup. Finally, we compare the uptake of IgG- vs. C3b-coated beads and highlight that the latter happens with lower efficiency.

## Results

### Purified C3b triggers phagocytosis and killing of *E. coli* by human neutrophils via complement receptor 1

**Figure 1:**
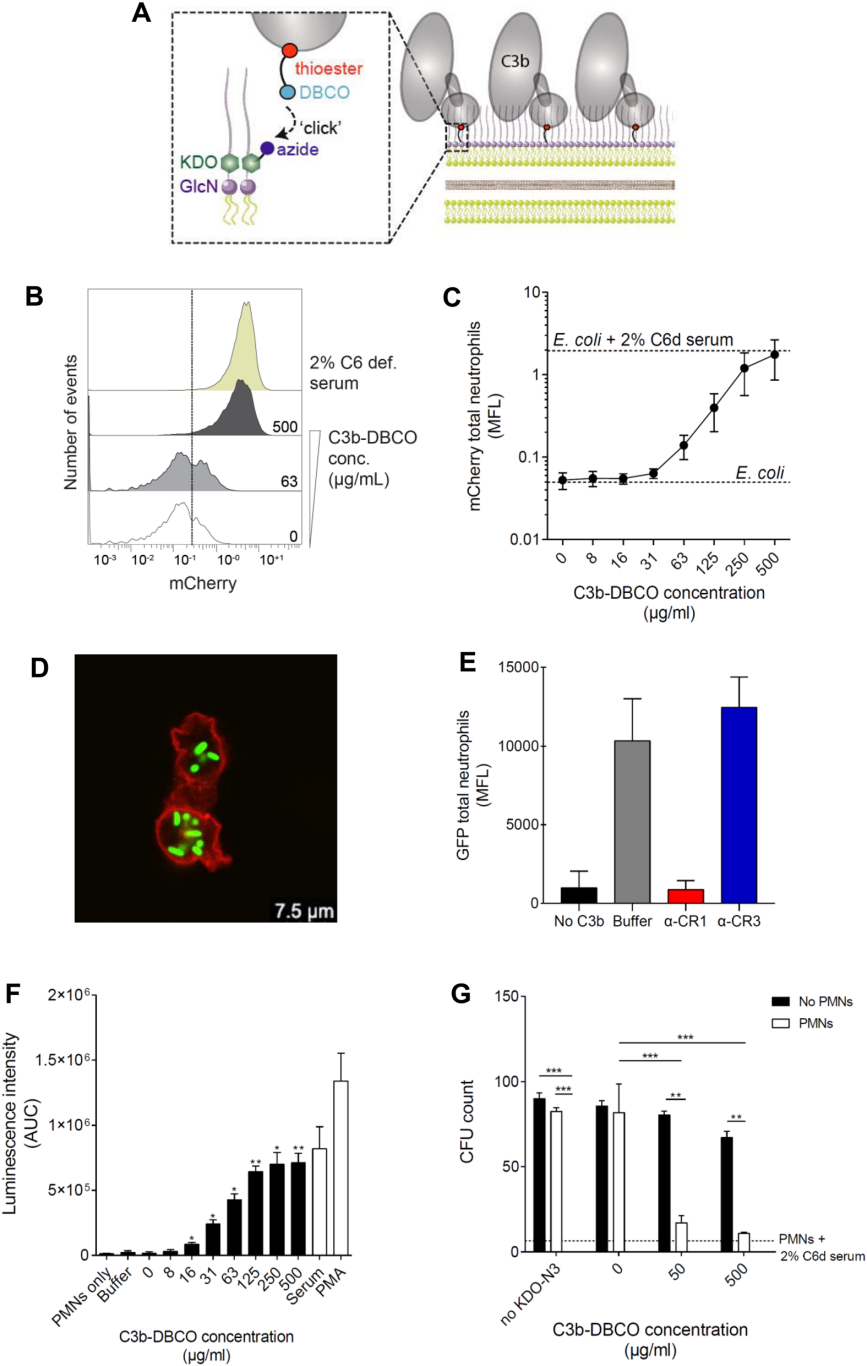
C3b-*E. coli* directly induce phagocytosis and killing by human neutrophils. (**A**) To produce C3b-*E. coli*, the thioester of purified C3b was reacted with DBCO-PEG_4_-maleimide to produce C3b-DBCO. Then bacterial LPS decorated with KDO-N3 was reacted with C3b-DBCO to produce C3b-*E. coli*. (**B**) Flow cytometry histograms displaying the mCherry intensity distribution of engulfed C3b-*E. coli* in the neutrophil population, for each concentration of C3b-PEG_4_-DBCO. In *yellow E. coli* was labelled with 2% C6-deficient serum (to prevent MAC formation). (**C**) C3b-DBCO dose-dependent increase of mCherry MFL in neutrophils. Mean ± SEM of 5 independent experiments. (**D**) Representative confocal microscopy image of phagocytosed C3b-*E. coli* labelled with 190 µg/mL of C3b-DBCO. red = wheat germ agglutinin Alexa-647, (lectin binding β-1,4-*N*-acetylglucosamine carbohydrate residues on membranes), green = GFP-expressing MG1655. (**E**) Inhibition of the interaction of GFP C3b-*E. coli* with neutrophils by anti-CR1 (red) and anti-CR3 (blue) blocking antibodies in terms of GFP MFL of internalized C3b-*E. coli* of the total neutrophil population. Mean ± SEM of 3 independent experiments. (**F**) Chemiluminescence intensity of reactive oxygen species released by neutrophils after incubation with C3b-*E. coli* MG1655 labelled with increasing concentrations of C3b-DBCO. Stimulation with 2.7 µM phorbol myristate acetate (PMA), a potent non-receptor initiator of endogenous ROS production, included as a benchmark. *E. coli* opsonized with 2% C6-deficient serum (to prevent MAC formation) was also included as positive control. Measurement expressed as mean area under the curve (AUC) ± standard deviation (SD) of 4 independent experiments. Statistical analysis was done using one-way ANOVA with a Dunnett correction for multiple comparisons and displayed only when significant compared to control “0 µg/mL C3b-DBCO” as * (p ≤ 0.05) and ** (p ≤ 0.01). (**G**) CFU count of bacteria labelled with 0, 50, or 500 µg/mL C3b-DBCO, after co-incubation with (“PMNs”) or without (“No PMNs”) neutrophils. CFU count of unlabelled bacteria (with neither KDO-N_3_ nor C3b-DBCO) opsonized with 2% C6-deficient serum (dashed line) is a positive control mimicking the natural deposition of complement. The graph represents the mean of CFU count ± SEM of 3 independent experiments. Statistical analysis was done using a two-way ANOVA with a Dunnett correction for multiple comparisons and displayed only when significant as **(p ≤ 0.01) and ***(p ≤ 0.001).

To study whether C3b can directly trigger phagocytosis of bacteria in the absence of other serum opsonins, we developed a surface chemistry method to site-specifically couple C3b onto the outer membrane of *Escherichia coli* (*E. coli*) (Fig. 1A). In short, lipopolysaccharides (LPS) were labelled via metabolic incorporation of KDO- N_3_ by culturing fluorescent bacteria in a medium containing azide-modified keto-deoxy-octulosonate (KDO), a major component of LPS [33]. Subsequently, C3b was coupled to metabolically incorporated KDO-N_3_ via click chemistry^32,33^ (S2A). To this end, the thioester domain of C3b was site-specifically labelled with a maleimide linker^34^ containing a dibenzocyclooctyne (DBCO) group. DBCO is the bioorthogonal partner of azide that allows covalent coupling in the absence of copper, which is compatible with living organisms^32,33^. The fact that the DBCO group was bound to the thioester domain allowed us to label C3b molecules onto bacteria in their natural orientation, thus preserving the accessibility of binding sites essential for recognition by immune-cell receptors.

To study phagocytosis, we incubated C3b-labelled *E. coli* (denoted C3b-*E. coli*) with human neutrophils, which are the first cells recruited to sites of infection to engulf and kill invading pathogens. We observed that coupling C3b to *E. coli* increased the association of bacteria to neutrophils (determined by either measuring the mean fluorescence mCherry intensity (Fig. 1B,C) or the % of mCherry-positive neutrophils (S2B). Interaction of cells with bacteria prepared with the highest concentration of C3b-DBCO is comparable to the benchmark set by the physiologically opsonized bacteria (2% human serum devoid of complement protein C6 to prevent lysis) (Fig. 1C). Using confocal microscopy, we observed that C3b-coated bacteria were indeed internalized (Fig. 1D).

Next, we investigated the receptors involved in the phagocytosis of C3b-*E. coli* by neutrophils by pre-incubating neutrophils with either CR1 or CR3 blocking antibodies (α-CR1 and α-CR3). While CR3-blocking antibodies did not affect phagocytosis of C3b-*E. coli*, we observed that CR1-blocking antibodies potently reduced phagocytosis (Fig. 1E). Inhibiting CR1 reduced both the absolute number of bacteria associated with neutrophils (Fig. 1E) as well as the percentage of GFP-positive neutrophils (S2C). Also, in confocal microscopy, we observed that α-CR1, but not α-CR3, prevented phagocytosis of C3b-*E. coli*.

Finally, we examined whether CR1-mediated uptake of C3b-*E. coli* led to intracellular killing.

During phagocytosis, bacteria are enclosed into the phagosome, which fuses with lysosomes to create a degradative environment for the enclosed target. A hallmark of this phagolysosomal stage is the assembly of the nicotinamide adenine dinucleotide phosphate (NADPH) oxidase, an enzymatic complex that greatly contributes to pathogen killing by producing toxic reactive oxygen species (ROS)^35^. First, we assessed whether uptake of C3b-*E. coli* induced this oxidative ‘burst’ using the well-established chemiluminescence assay using the ROS-sensitive indicator luminol^36^. C3b-*E. coli* was incubated with neutrophils and ROS production was measured continuously for 90 min (Fig. 1F). These measurements revealed that C3b-*E. coli* stimulated the production of ROS in a dose-dependent manner, suggesting a key role of C3b in the phagolysosomal processing of these bacteria. Remarkably, the height of the ROS plateau is comparable to the level of ROS production observed during phagocytosis of serum-opsonized bacteria. Finally, we determined whether the neutrophils kill C3b-*E. coli* by enumerating surviving bacteria. Consistent with the fact that C3b induced ROS formation, we observed that C3b mediated the killing of *E. coli* by neutrophils (Fig. 1G).

In summary, these results demonstrate that C3b can directly drive phagocytosis and killing of *E. coli* in the absence of other serum components.

### Time-dependent cell morphology and basic mechanical features of C3b- and IgG-mediated phagocytosis are indistinguishable

Having established that purified C3b can drive phagocytosis in neutrophils, we further studied the uptake mechanism. In the seventies, Kaplan et al. introduced the idea that IgG-opsonized targets induced protruding pseudopods (“zipper model of phagocytosis”) whereas complement-opsonized targets, in which iC3b was considered determinant, were sinking without any pseudopod formation (“sinking model of phagocytosis”)^30^. Although the results of the Kaplan report have since been disputed^37,38^, more recent single-live-cell studies have provided evidence that phagocytosis via antibodies can be different from serum-opsonized particles^5,6^. However, these studies were performed with particles that not only have C3b on their surface, but also C4b, iC3b, and, in some cases, IgGs^39^.

**Figure 2:**
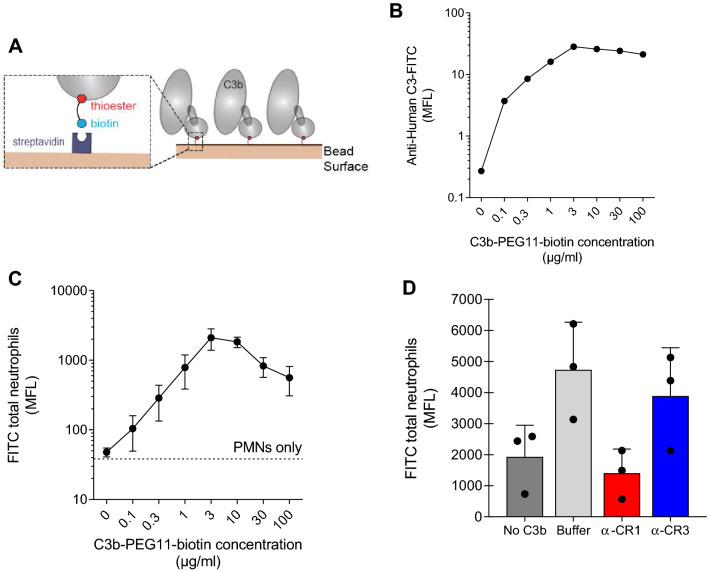
C3b-labelled beads directly interact with human neutrophils via complement receptor 1. (**A**) Illustration of the labelling method to produce C3b-beads. FITC-labelled 2.8-µm streptavidin beads incubated with increasing concentrations of C3b-PEG_11_-biotin. (**B**) Dose-dependent increase of FITC MFL confirming the deposition of C3b-biotin on beads. The graph represents one experiment. (**C**) Dose-dependent increase of FITC MFL in neutrophils dependent on the C3b-biotin concentration used to opsonize beads. The graph represents the mean ± SEM of 3 independent experiments. The dashed line indicates the base fluorescence of neutrophils. (**D**) Inhibition of the interaction of FITC and C3b-labelled beads with neutrophils by anti-CR1 (red) and anti-CR3 (blue) blocking antibodies in terms of decrease of FITC MFL in the total neutrophil population. Mean ± SEM of 3 independent experiments.

Here we combine our methods to prepare C3b-coated targets with flow cytometry and single-live-cell/single target assay^5,40^ to study C3b-mediated phagocytosis. Since polystyrene beads are more suitable for our dual-micropipette experiments^5,40^ than bacteria, we first coupled C3b to beads with the thioester domain labelled with biotin^34^ (Fig. 2A,B). Indeed, we observed that beads associate with neutrophils in a C3b dose-dependent manner (Fig. 2C, S3A). Consistent with the C3b-*E. coli* data, we observed that the association of C3b-beads to neutrophils was blocked by antibodies blocking CR1, but not CR3 (Fig. 2D, S3B).

**Figure 3:**
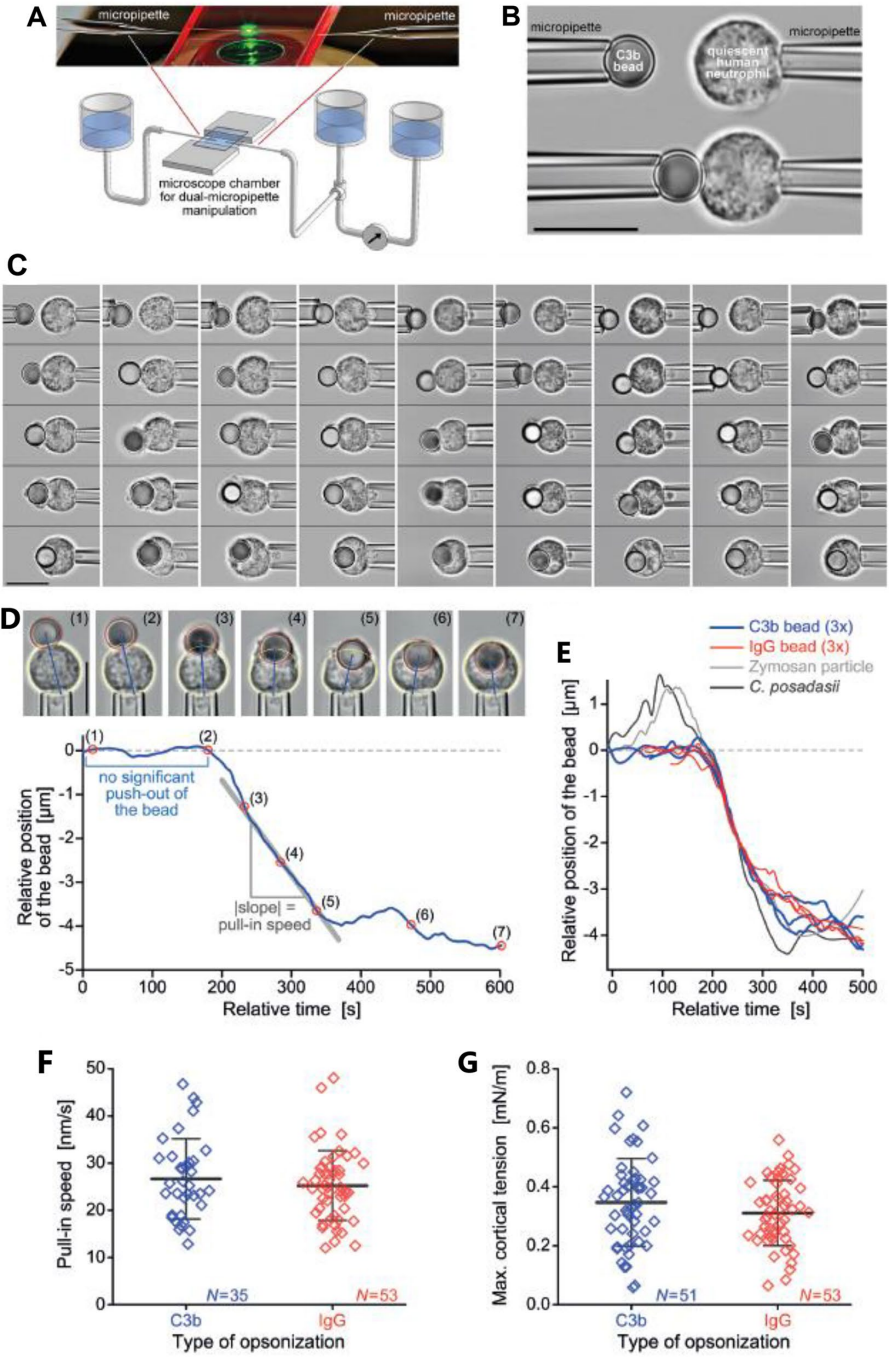
Single-live-cell/single-target assay to examine phagocytosis of C3b-coated beads by human neutrophils and quantitative analysis of biomechanical parameters. (**A**) Dual-micropipette manipulation setup. Two facing glass micropipettes with inner tip diameters of ~ 2 to 3 µm are inserted into a microscope chamber made from sandwiched coverslips. The pipette-aspiration pressure is controlled by height adjustments of the connected water reservoirs. A reference reservoir and differential pressure transducer allow us to monitor the aspiration pressure of the cell-holding pipette (on the right) throughout the experiment. (**B**) Two pipettes pick up and hold a C3b-coated bead and passive human neutrophil with gentle suction and bring them into contact. (**C**) Qualitative inspection of the phagocytosis of C3b-beads by human neutrophils. Vertical filmstrips of nine example experiments (selected from 51 experiments with C3b-beads) depict typical neutrophil morphology from the first contact with the target bead to the completion of engulfment (Movie S1). All scale bars denote 10 µm. (**D**) Measurement and analysis of the bead trajectory. We match a circle (red) and an ellipse (yellow) to the bead and main cell body, respectively, and calculate the distance from the bead center to the facing far edge of the cell body (blue straight lines). The bead positions are recorded relative to the initial configuration established after bead attachment but before phagocytosis. A representative bead trajectory (blue curve) depicts the bead positions as a function of time. Numbered symbols (red open circles) along this trajectory mark the time points at which the respective images were taken. The pull-in speed is determined as the slope of a straight-line fit to a suitable segment of the bead trace. The scale bar denotes 10 µm. (**E**) Example traces of target particles during phagocytosis by human neutrophils. Three typical trajectories of C3b-beads (blue) are shown together with three trajectories of beads coated with polyclonal anti-BSA rabbit IgG (red). Also included are previously measured traces of a zymosan particle^5^ and a *C. posadasii* endospore^41^. (See also Movie S2). (**F**) Summary of measurements of the pull-in speeds of C3b- and IgG-beads. (**G**) Summary of measurements of the maximum cortical tensions of neutrophils during phagocytosis of C3b- and IgG-beads. Error bars in F and G show the standard deviation from the mean.

Next, we employed single-live-cell imaging to examine the time-dependent behavior of human neutrophils during one-on-one encounters with C3b-beads. In each dual-micropipette experiment, we brought a non-adherent, initially passive neutrophil into contact with a target bead (Fig. 3A,B) and recorded time-lapse videos of the ensuing cell response (Fig. 3C, Movie S1). These single-cell experiments confirmed that human neutrophils readily recognize C3b-coated targets upon contact. C3b-beads consistently drew a strong adhesive and phagocytic response by neutrophils. The typical engulfment time, estimated from the first visible cell deformation triggered by the captured bead to the closure of the phagocytic cup, was approximately 1–2 min. In many (but not all) experiments, the overall cell shape remained roughly axisymmetric throughout the engulfment process, and the bead stayed within, or close to, the microscope’s focal plane, allowing us to visualize the time-dependent cell morphology in exceptional detail (Fig. 3C). The typical cell deformation consisted of the formation of a phagocytic cup that advanced around the bead without significantly pushing the bead itself outwards, followed by the inward motion of the bead as the main cell body gradually resumed a spherical shape (Movie S1). The same phagocytosis morphology—lacking an initial outward push of the target—previously had been observed during antibody-mediated phagocytosis^5,40^. Indeed, when we repeated the current single-cell experiments with IgG-coated beads that had a similar size (5 µm) as the C3b beads, we were unable to detect obvious morphological differences between the phagocytic uptake of C3b- and IgG-coated beads (Movie S2). In contrast, serum-opsonized targets such as zymosan particles^5^ and fungal pathogens^41^ previously were found to experience a noticeable outward displacement at the onset of phagocytosis experiments, underlining the discriminative power of this single-cell assay.

In addition to the above qualitative inspection of the phagocytosis morphology, we quantified key biomechanical aspects of target internalization, such as the trajectory of the target bead and the neutrophils’ cortical tension during engulfment. Quantifying these features allows us to assess how the neutrophil remodels its cytoskeleton to achieve the deformations accompanying phagocytosis.

Typical example trajectories of C3b-beads during phagocytosis are shown in Fig. 3D,E (blue curves), depicting the recorded bead positions as a function of time. The resulting bead traces allow us to quantify the amount of initial target push-out and the speed at which each particle is pulled into the cell (Fig. 3D). The shown trajectories confirm that C3b beads did not experience a significant outward push at the onset of phagocytosis, in contrast to the typical motion of serum-opsonized zymosan particles^5^, which traditionally have been considered to be prototypical targets for the study of complement-mediated phagocytosis (Fig. 3E). We also have included in Fig. 3E a representative trajectory of an endospore of the fungus *Coccidioides posadasii*^41^, illustrating that the initial target push-out by neutrophils is common to serum-opsonized zymosan and fungi. On the other hand, considering natural cell-to-cell variability, the trajectories of C3b beads were indistinguishable from those of IgG-coated beads (*red curves* in Fig. 3E; Movie S2). This similarity bears out in the detailed analysis of the pull-in speeds (defined in Fig. 3D) of these two types of target bead. Our comparison of the speeds measured in *N* = 35 experiments with C3b beads and *N* = 53 experiments with IgG beads revealed no significant difference (Fig. 3F).

Finally, we also compared the maximum cortical tensions during the uptake of C3b- and IgG-coated beads. The cortical tension is a measure of the cell’s resistance to expansion of its apparent surface area. An initially round cell inevitably increases its surface area during phagocytosis. The resulting rise of the cortical tension reflects the mechanical effort expended by the cell during this process. In our experiments, the cell first tries to recruit part of the needed surface area by retracting its projection from inside the holding pipette. The operator prevents this by increasing the pipette-aspiration pressure, aiming to keep the projection length constant. We recorded the aspiration pressure throughout each experiment and converted it to an estimate of the cortical tension using a well-known mathematical relationship based on Laplace’s law^42^. This analysis did not find a significant difference between the maximum tensions measured during the phagocytosis of C3b beads (*N* = 51) and IgG beads (*N* = 53) (Fig. 3G).

In summary, our highly discriminatory single-live-cell assay has been unable to discern differences between the immunomechanical responses of human neutrophils to C3b- and IgG-coated beads. Thus, neutrophils appear to utilize the same machinery of phagocytosis mechanics during interactions with these two types of targets. We, therefore, speculate that downstream of IgG- and C3b-specific receptor recognition and signaling, the respective biochemical response paths partially merge to induce a common mechanical cell behavior.

### Internalization of C3b-beads is less efficient than IgG-beads

**Figure 4:**
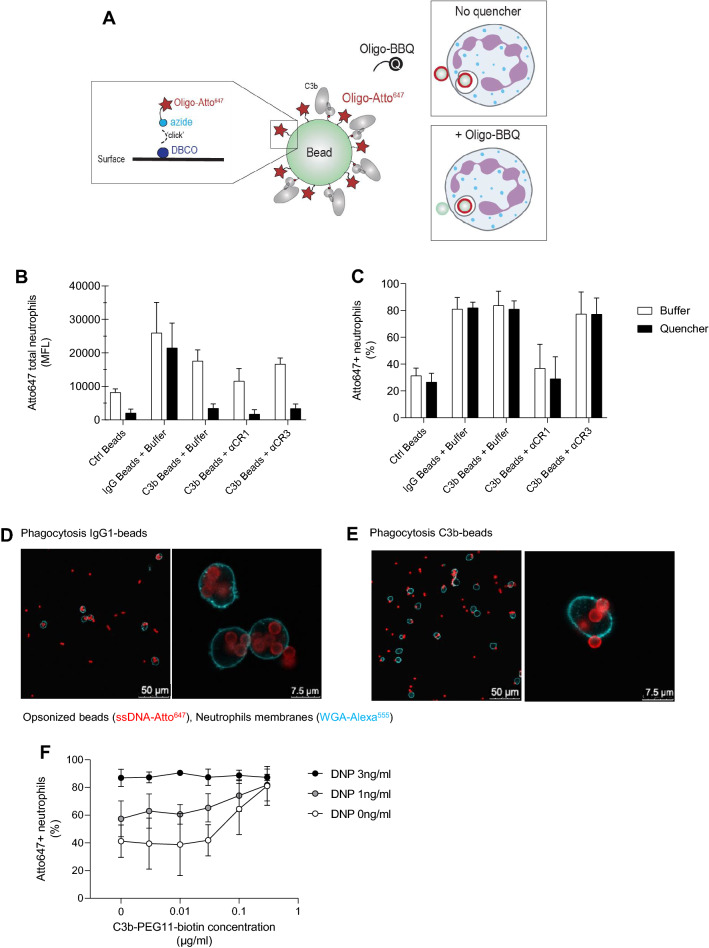
Quenching assay of C3b-beads vs. IgG1-beads by human neutrophils. (**A**) Illustration of the method used to label beads for the quenchable phagocytosis assay. Beads are reacted with DBCO-NHS, which covalently binds the azide-functionalized oligomer fluorescently labelled with Atto647 “*Oligo-Atto647*”. The fluorophore is quenched by hybridization with the complementary oligomer bound to BlackBerry Quencher (BBQ). (**B,C**) Uptake of empty, IgG- and C3b- beads evaluated in the presence (black) or absence (white) of the quencher. Results are expressed in terms of: (**B**) mean Atto647 fluorescence intensity of the total neutrophil population and (**C**) percentage of Atto647-positive neutrophils in the total population. (**B,C**) represent the Mean ± SD of 3 independent experiments. (**D,E**) Confocal microscopy images of a representative phagocytosis assay of Atto647-beads incubated in the presence of (**D**) 3 µg/mL IgG1 anti-DNP or (**E**) 1 µg/mL C3b-biotin. Staining information: red = ssDNA Atto647, (fluorescently-labelled single-stranded DNA oligonucleotide), blue = Wheat Germ Agglutinin-Alexa fluor 555 (lectin binding β-1,4-N-Acetylglucosamine carbohydrate residues on membranes). (**F**) Uptake of beads opsonized with increasing concentrations of combined anti-DNP IgG and C3b-biotin expressed as percentage of Atto647-positive neutrophils in the total population. Mean ± SD of 3 independent experiments.

Although the above single-live-cell assays did not reveal morphological differences between phagocytosis of C3b- and IgG-coated targets, it was still unclear whether phagocytosis of these targets occurred with the same efficiency. In micropipette experiments, it is difficult to assess the efficiency of uptake since the target is artificially held in contact with the neutrophil by the operator. Therefore, we extended the bulk internalization assay with DNA-based quenching^43^. Briefly, target beads were labelled with an Atto647 dye that is coupled to a single-stranded oligonucleotide. The fluorescence of non-internalized beads can be quenched by counter-staining with a BlackBerry Quencher (BBQ) that is coupled to the reverse complementary oligonucleotide. The unquenched condition provides information regarding the interaction of neutrophils with C3b-beads (adherence + internalization), while in the quenched condition only the fluorescent signal of internalized beads is present (Fig. 4A, S4).

We first studied the internalization of IgG1-labelled beads. Oligo-Atto647-beads were labelled with DNP-PEG-biotin and human anti-DNP IgG1^44^. Next, IgG-labelled beads were incubated with human neutrophils for 10 min before adding the quencher. We observed that the quencher marginally affected the Atto647 fluorescence of the neutrophil population (Fig. 4B,C), indicating that most IgG-beads had been internalized, a conclusion that was supported by confocal microscopy (Fig. 4D).

Next, we optimized the labeling of Atto647 beads with C3b (S5A) and evaluated their internalization. In contrast to IgG-beads, we observed that the quencher significantly reduced the Atto647 fluorescence of neutrophils incubated with C3b-beads, suggesting that only a fraction of C3b-beads was internalized (Fig. 4B). In contrast, the percentage of neutrophils interacting with beads was minimally affected by the quenching treatment, confirming that most cells interacting with C3b-beads also internalized at least one bead (Fig. 4C). This result was confirmed by confocal microscopy, in which externally adherent beads were found on the cell surface (Fig. 4E). In accordance with previous results, the internalization of C3b-beads to neutrophils was impaired upon CR1-blocking treatment, confirming the important role of CR1 in mediating the interaction between neutrophils and C3b targets (Fig. 4C).

Finally, we investigated the uptake of beads opsonized with combinations of IgG and C3b to verify cooperation between the two opsonin in enhancing uptake. The work of Ehlenberger and Nussenzweig previously showed that IgG and C3b taken singularly are poor opsonins, but display a synergistic effect when used in combination to opsonize erythrocytes^45^. After demonstrating that the two labelings do not interfere with each other (Fig. S5B, C), we opsonized beads with increasing concentrations of IgG and C3b and verified uptake. Beads labelled with C3b + 1 ng/mL of DNP, as docking antigen for IgG1, displayed a higher uptake with respect to beads labelled with the same concentration of C3b and IgG alone (Fig. 4F). At higher concentrations a plateau of phagocytosis was reached that was not perturbed by varying C3b concentration.

In all, our quenching method shows that although C3b coupling to beads leads to a strong CR1-dependent interaction with the cell membrane, only a fraction of adherent C3b-beads is internalized. In contrast, neutrophil interaction with IgG-beads leads to internalization. When beads are opsonized with both opsonins, C3b can enhance phagocytosis of IgG-labelled beads and vice versa.

### Neutrophils spread slower on C3b-coated surfaces in frustrated phagocytosis assays

**Figure 5:**
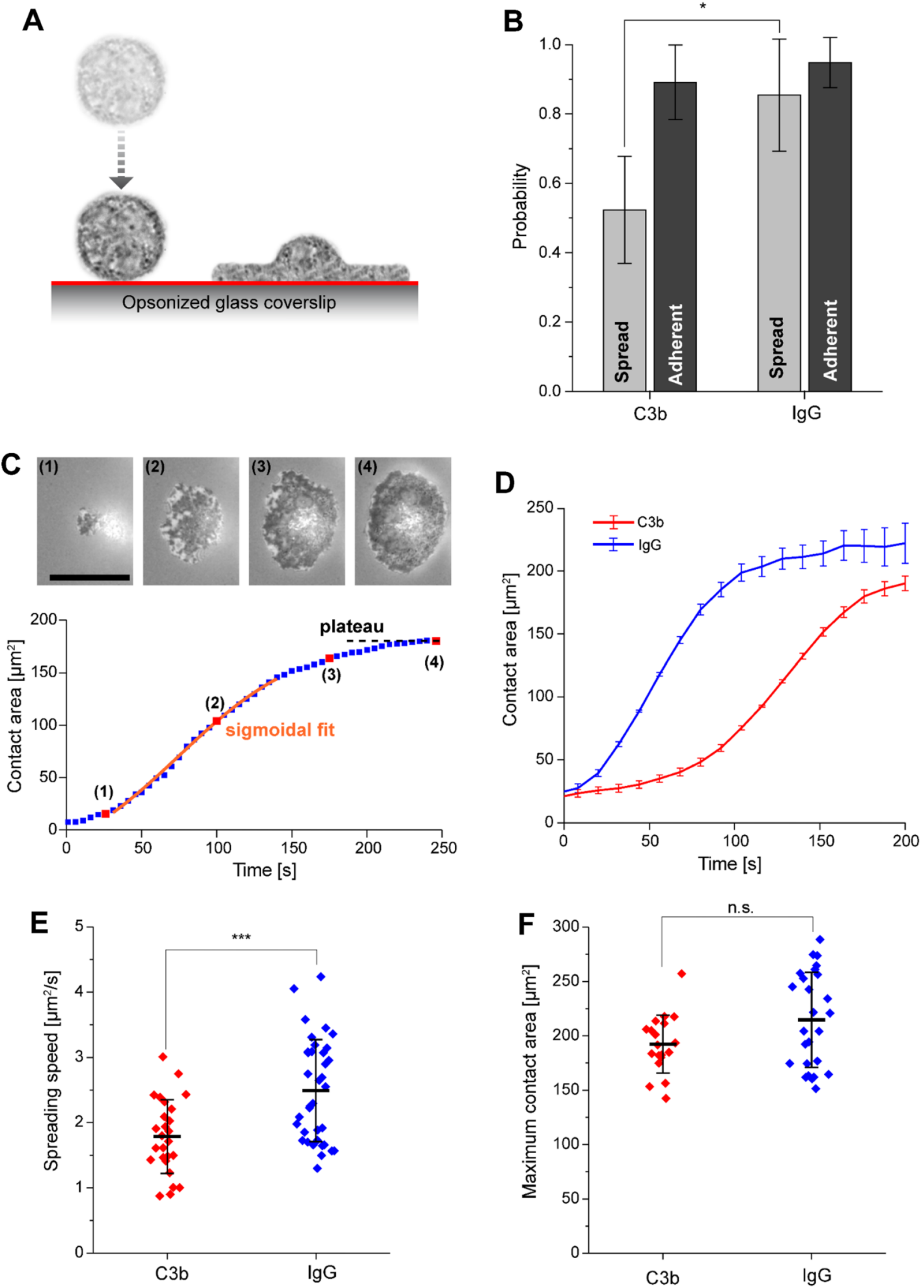
Frustrated phagocytosis of C3b vs. IgG coated surfaces by human neutrophils. (**A**) Illustration of frustrated phagocytic spreading assay. (**B**) Spreading probability and adhesion probability on C3b (4 trials, 586 total cells) vs. IgG-coated coverslips (5 trials, 967 total cells). (**C**) Sample images of the contact region of a neutrophil spreading on IgG (scale bar denotes 10 μm), with the corresponding curve of contact area vs. time. The spreading speed was determined using a sigmoidal fit, and the maximum contact area was measured by taking the time average of the contact area at the plateau of the spreading curve. (**D**) Mean contact area vs. time for cells spreading on C3b (*N* = 26) vs. IgG (*N* = 36). (**E**) Spreading speed is significantly lower on C3b-coated surfaces compared to spreading on IgG. *N* = 26 for C3b, *N* = 36 for IgG. (**F**) Maximum contact area is not significantly different on C3b (*N* = 19) vs. IgG (*N* = 26). Error bars in (**D**) show standard error of the mean and error bars in (**E,F**) show standard deviation. A two-tailed t-test was used to test for significance in (**B,E,F**) with significance indicated by *(p ≤ 0.05) or ***(p ≤ 0.001).

Having found that C3b-coated beads are phagocytosed much less than IgG-coated beads, we sought to determine whether this difference could be due to a change in cell spreading dynamics in response to C3b vs. IgG. To specifically probe the speed and the extent of spreading, we utilized a frustrated phagocytosis assay in which neutrophils were exposed to glass coverslips coated with similar densities of either IgG or C3b (Fig. 5A, S6).

After depositing human neutrophils onto these surfaces, we monitored the time course of spreading by imaging the contact region between individual cells and the coverslip with high resolution using reflection interference contrast microscopy (RICM). First, we quantified the total fraction of cells that adhered and/or spread over a 30-min time window. In agreement with the bulk phagocytosis assays, we found that while most cells adhered to either surface, the fraction of cells that spread was significantly smaller on C3b-coated than on IgG-coated surfaces (Fig. 5B).

We then assessed spreading dynamics by analyzing the contact area of individual spreading cells as a function of time (Fig. 5C). Plots of the average contact area versus time indicate that neutrophils spread slower on C3b-coated than IgG-coated surfaces (Fig. 5D). Indeed, our analysis of the spreading curves revealed that the mean cell spreading speed on C3b was significantly lower than on IgG (Fig. 5E). On the other hand, the extent of spreading as indicated by the maximum contact area appeared to be similar for both conditions (Fig. 5F). This implies that the difference in target internalization observed in the bulk phagocytosis assay is not due to a difference in the extent of phagocytic spreading, but instead appears to be a consequence of a lower likelihood of cells initiating spreading on C3b-coated surfaces.

## Discussion

In most if not all physiological situations where microbes invade a human host, complement components participate in the first line of the host’s immune defense. These molecules in serum can mount several types of attack, including the permeabilization of bacteria, the production of chemoattractant peptides, and the opsonization of microbial surfaces. Although other, more specific forms of immune recognition recently have dominated immunological research, it is worth noting that in many cases, complement-based mechanisms precede, or participate in, the respective response programs^1^. For example, the initiation of specific antibody or T cell responses often requires the phagocytic uptake of microbes, which is far more effective if the microbes are opsonized with complement components^39^.

This work’s focus on C3b-mediated phagocytosis by human neutrophils has resulted in interesting insights into a little-studied but, as we show, potentially important mechanism of target uptake. Past investigations of complement-mediated phagocytosis mainly have examined interactions between iC3b and CR3, whereas reports about C3b- and CR1-mediated phagocytosis have been scarce. The traditional interest in iC3b in the context of phagocytosis is certainly reasonable. CR3, the main receptor of iC3b, is a β_2_ integrin that is well known to be involved in cell adhesion and migration, which both are essential functional elements of immune-cell phagocytosis^46–54^. In contrast, most existing phagocytosis research on C3b has considered this complement fragment as a transient intermediate that is swiftly converted to iC3b. Furthermore, surface-bound C3b drives the alternative pathway amplification loop and initiates the terminal pathway, which forms the highly potent chemoattractant C5a, and the lytic membrane attack complex. The downregulation of these C3b-mediated effector functions, and the transport of C3b-coated immune complexes, are among the best-known functions of the C3b/C4b receptor CR1. Given this well-established regulatory role at a pivotal point of the complement-activation cascade, it is perhaps not surprising that a possible function of CR1 as a phagocytic receptor has received less attention.

On the other hand, several past studies suggest that surface-bound C3b also can stimulate immune cells directly. A relatively small number of studies has actually inspected C3b-mediated phagocytosis by immune cells^27–29,45,55–58^. Generally, these studies supported a possible role of C3b as a ligand that enhances immune adherence of opsonized particles in the vicinity of cells, facilitating their phagocytosis via other mechanisms mediated by iC3b or IgG. In addition, they often used targets coated with serum or introduced additional stimuli that could bias the phagocyte’s response. To our knowledge, the present work is the first in-depth study of purely C3b-mediated phagocytosis by human neutrophils. Contrarily to the results of the present paper, in their work Ehlenberger and Nussenzweig do not observe phagocytosis when erythrocytes are opsonized by IgM+ early classical pathway components + C3b/iC3b and conclude that C3b/iC3b provide an additional stimulus to IgG, probably enhancing the adherence of particles. One possible explanation lies in the use of erythrocytes as C3b targets. In fact, erythrocytes display CR1 on their surface, possibly influencing the outcome of assays. In general, given the discrepancy observed between the ready internalization of *E. coli* and the weaker internalization of beads, it is worth considering an additive effect of stimuli on the bacterial membrane aiding C3b/iC3b-mediated phagocytosis. This is especially relevant when no antibodies are present, for example at the first encounter with the pathogen.

Our study was enabled by a reductionist, interdisciplinary approach employing C3b-coated bacteria and beads. Benefitting from recent advances in chemical metabolic labeling of bacteria, we site-specifically labelled C3b and coupled it to *E. coli* bacteria in the proper orientation. This method allowed us to assess the ability of pure C3b to drive the phagocytosis of *E. coli*. In addition, our experiments with C3b-coated bacteria showed that later steps of phagosome maturation were functional for these targets. The C3b-mediated phagocytic uptake of *E. coli* not only induced a pronounced oxidative burst but also resulted in the efficient killing of the bacteria in a dose-dependent manner. This agrees with a previous study in which neutrophil phagocytosis of C3b-coated yeast particles triggered a robust oxidative burst^29^. The importance of our findings is supported by ample evidence that serum-opsonized targets can display not only iC3b, but also a substantial fraction of C3b^59,60^. For example, serum incubation of various microbes resulted in the deposition of C3b:iC3b ratios of roughly 1:7 onto yeast, 5:7 onto *Staphylococcus aureus*, and as much as 2–3:1 onto *E. coli*, *Haemophilus influenzae*, *Streptococcus pneumoniae*, and *Streptococcus pyogenes*^60^.

Our work identified CR1 as the main receptor directly involved with C3b-driven particle uptake. Although our results prove that a direct path from CR1 engagement to phagocytosis exists, it is yet unknown how ligation of CR1 leads to the phagocytic uptake of C3b-coated targets. The fact that even our highly discriminatory single-live-cell assay could not distinguish between C3b- and IgG-mediated phagocytosis at any stage of the neutrophil response suggests that the signaling mechanisms initiated by CR1 and FcγRs may converge at some point, at least as far as control of the cells’ mechanical behavior is concerned. Although it remains unclear where these paths might merge, we note that multiple studies have indicated a cooperative role of CR1 and FcγRs^61,62^ or reported the colocalization of these receptors^63,64^, suggesting the possibility that CR1 signals through the immunoreceptor tyrosine-based activation motif (ITAM) domains of adjacent Fcγ receptors. CR1 engagement also has been shown to lead to phospholipase D activation^21^, as does FcγR engagement^65^. Furthermore, both CR1^66^, as well as FcγRs^49,67,68^, can cooperate with CR3. Further studies of receptor crosstalk and the signaling paths activated by these receptors are needed to sort out the molecular mechanisms governing C3b-mediated phagocytosis.

Earlier findings established the idea that complement- and IgG- mediated phagocytosis displayed distinguishing morphological features, however these observations have been recently questioned and extended^37,38^. In accordance with these revisions, our single-live-cell comparison of C3b- and IgG-mediated phagocytosis suggests that CR1 ligation does not cause the distinct phagocytosis morphology observed previously between complement- and antibody-mediated phagocytosis^5,30,31,41^. It is important to note that earlier findings were obtained under very different conditions. Unlike the present live-cell study of purely C3b-mediated phagocytosis by human neutrophils, Kaplan^30^, like many others, examined mouse macrophages that had been fixed during the engulfment of serum-opsonized sheep erythrocytes. Notably, mouse peritoneal macrophages F4/80^+^ do not express CR1, thus limiting the study of complement-mediated uptake to CR3 engagement only^69^. The single-live-cell assay used in the present study visualizes the time-dependent cell behavior with unique clarity, and our findings are supported by large numbers of observations. This approach gives us high confidence that human neutrophils do not exhibit significant morphological differences during the phagocytic uptake of C3b- and IgG-coated targets. Our single-live-cell comparison of C3b- and IgG-mediated phagocytosis provides other intriguing clues about the cellular processing of C3b-recognition events by CR1. Our results suggests that CR1 ligation does not cause the distinct phagocytosis morphology previously observed during the uptake of serum-opsonized zymosan and *C. posadasii* particles (Fig. 3E)^5,41^. According to the mechanistic explanation of differences between IgG-mediated phagocytosis and the uptake of opsonized zymosan^6^, the similarity between IgG- and C3b-mediated phagocytosis suggests that structural linkages between the neutrophil membrane in contact with either target type and the neutrophil cytoskeleton exhibit similar strengths.

Although our single-live-cell experiments showed that C3b drives a strong CR1-mediated interaction of beads with neutrophils (with no obvious biomechanical differences compared to IgG), our bulk assays demonstrate that C3b-induced phagocytosis is less efficient than IgG. C3b induced strong CR1-mediated interaction of beads with neutrophils (in bulk assays); however, engulfment of C3b beads was comparatively poor. This observation was confirmed by the frustrated phagocytosis assay, in which all cells successfully adhere to C3b-coated surfaces, but only a fraction spreads on the cover glass as to engulf the target. This suggests that the single-cell/single-target technique might skew the process towards a productive interaction, for example, by forcing longer and stronger contacts between cell and target. Finally, we cannot exclude that the manipulation of neutrophils is at least partially responsible for the differences we observed between techniques. Non-forced C3b-CR1 interactions in frustrated phagocytosis assays lead to slower spreading of neutrophils on C3b surfaces compared to the benchmark opsonin IgG, interacting with FcγRs. This observation suggests that C3b binding to CR1 generally triggers a less rigorous response than IgG binding FcγRs, which might explain differences observed in bulk experiments.

In contrast to beads, we observed strong CR1-mediated internalization of *E. coli*-C3b by neutrophils. We can speculate on the differences between phagocytosis of C3b-*E. coli* and C3b-beads. Components of the bacterial outer membrane could act as a second stimulus interacting with innate immune receptors of neutrophils. Alternatively, the differences could be due to the varying density and distribution of C3b moieties achieved on *E. coli* versus beads. Since C3b was coupled to LPS in biological membranes this confers C3b a certain degree of lateral diffusion that could aid receptor engagement and activation. Potentially, a higher local density of C3b might also better represent the physiological situation in which foci of C3b deposit in the surrounding of the C3b convertases. Indeed, CR1 has been shown to have a much higher affinity for multimeric C3b compared to monomeric C3b^70^, which could explain why we observe a strong neutrophil response to densely coated targets.

In conclusion, we have shown that C3b plays a role in phagocytosis by neutrophils. Although C3b is less efficient than IgG in mediating target internalization, complement opsonization by C3b alone is sufficient to induce engulfment and killing of *E. coli*, indicating that iC3b is not the sole complement opsonin responsible for phagocytosis. These data provide further insights into our understanding of complement-mediated phagocytosis.

## Materials and methods

### C3b purification and modification

C3 was purified from fresh serum, converted to C3b, and site-specifically biotinylated, as previously described^34^. Briefly, 1 mg/mL C3 in phosphate-buffered saline (PBS) was activated with 1.1 μg/mL trypsin for 10 min at 37 °C to expose its thioester, in the presence of 100 μg/mL of maleimide-PEG_11_-biotin linker. The reaction was then arrested by the addition of 5.5 μg/mL soybean trypsin inhibitor (SBTI) and 20 mM iodoacetamide, and the sample was incubated for 30 min on ice. C3b-biotin was finally purified by anion exchange chromatography and checked by SDS-PAGE and Western Blot. The generation of C3b-DBCO was conducted in the same way but using a maleimide-PEG_4_-DBCO linker (Jena Bioscience). The sample was purified, analyzed, and stored as described for C3b-PEG11-biotin.

### Neutrophil isolation

Two different methods were used to isolate neutrophils from human blood. Human neutrophils for bulk experiments were isolated from heparinized blood of healthy donors by a combination of density-gradient centrifugation and osmotic shock of red blood cells, as previously described^71^. On the other hand, because anecdotal evidence from past single-live-cell tests indicated that the osmolysis procedure might affect neutrophil longevity and phagocytic behavior (V. Heinrich*,* unpublished data), the neutrophils for our single-cell experiments were obtained by a different technique, as described previously^42^. In short, neutrophils obtained from whole blood of healthy donors were enriched by immunomagnetic negative selection using the EasySep Direct Human Neutrophil Isolation Kit (STEMCELL Technologies). This kit uses an isolation cocktail and functionalized magnetic beads to cross-link and magnetically remove cells other than neutrophils from the blood. After three 5-min magnetic separation cycles, the isolated neutrophils were resuspended in HBSS and gently rotated until use. For single-cell experiments, a small amount of cell suspension was introduced into the microscope chamber that contained HBSS with 1.26 mM Ca^2+^ and 0.9 mM Mg^2+^ (Thermo Fisher Scientific). Written informed consent was obtained from all subjects. The Institutional Review Board of the University of California Davis approved the protocol covering this study.

### Preparation of C3b-labelled *E. coli*

To develop the click chemistry-based C3b-labeling protocol an *E. coli* MG1655 strain expressing mCherry (pFCcGi was a gift from Sophie Helaine & David Holden^72^) was used. Bacteria were cultured in Luria broth with 50 μg/mL ampicillin at 37 °C until stationary phase. A 1:100 subculture of *E. coli* was then cultured overnight in the presence of ampicillin 50 μg/mL and 2 nM KDO-N_3_^34^ (Jena Biosciences). The incorporation of KDO-N_3_ in the LPS was verified by an overnight click incubation with DBCO-Alexa Fluor 488 (Jena Biosciences; Germany) detected by flow cytometer (MACSQuant VYB, Miltenyi Biotec; Germany). Serial dilutions of C3b-DBCO were incubated with KDO-N_3_-labelled bacteria at 4 °C for 24 h, to avoid bacterial growth and preserve C3b. C3b-DBCO labeling was detected with FITC-labelled F(ab’)2 Goat anti-Human-C3 by flow cytometry. A GFP-expressing *E. coli* MG1655 strain^73^ was labelled with C3b-DBCO as reported above and used for bulk experiments. Culture of this strain required 30 μg/mL gentamycin for plasmid maintenance.

### Preparation of C3b- and IgG-coated beads for single-cell experiments

C3b-coated targets for single-cell phagocytosis experiments were prepared by binding C3b-biotin to streptavidin-coated polystyrene beads (nominal diameter of 4.47 µm; Spherotech). After washing the streptavidin beads three times in Ca^2+^ and Mg^2+^-free Hank's Balanced Salt Solution –(HBSS) (Thermo Fisher Scientific) with 0.1% Tween-20 (Millipore Sigma), the beads were resuspended in HBSS without Tween and mixed with C3b-biotin solution at final concentrations of 100 µg/mL C3b-biotin and 3 × 10^6^ beads/mL. This suspension was placed on a rotator at 4 °C for 15 min and then kept at 4 °C until use.

IgG-coated microspheres were prepared as described previously^42^. In short, polystyrene beads with a 5.0 μm nominal diameter (Duke Standards Microspheres, Thermo Fisher Scientific) were incubated overnight at 4 °C in phosphate-buffered saline (PBS; IBI Scientific) containing bovine serum albumin (BSA; 10 mg/mL; AMRESCO). After three washes in PBS with 0.01% Tween 20, the beads were incubated with rabbit polyclonal anti-bovine albumin antibody (IgG fraction; Thermo Fisher Scientific) at room temperature for 1 h, or overnight at 4 °C. The beads were then washed three more times in the PBS/Tween solution and resuspended in PBS for storage at 4 °C until use.

### Preparation of C3b- and IgG1-coated beads for flow cytometry experiments

Dynabeads™ M-280 Streptavidin (Invitrogen) were magnetically washed with PBS with 0.05% human serum albumin (HSA) (Sigma-Aldrich). If beads were used in the phagocytosis assay, they were fluorescently labelled by incubating them for 30 min on ice with a solution of 0.5 mg/mL FITC fluorescein isothiocyanate (Sigma-Aldrich). After washing, a serial dilution of C3b-biotin was incubated with 1 × 10^8^ beads/mL for 1 h at 4 °C on a shaking platform. The excess C3b-biotin was washed, and beads were resuspended in RPMI 1640 + l-Glutamine (Gibco) + 0.05% HSA at a concentration of 1.875 × 10^7^ beads/mL and kept on ice until use.

The binding of C3b-biotin was assessed via a polyclonal FITC-labelled F(ab’)2 Goat anti-Human-C3 (Protos Immunoresearch) by flow cytometry (FACSVerse BD Biosciences). The maximum binding was reached when the beads were incubated with 3 µg/mL C3b-biotin.

For the quenching assay, we reprised the protocol of Lehmann et al.^43^. FITC-labelled Dynabeads M-280 Streptavidin (Invitrogen) were reacted for 2 h at room temperature with 100 µM DBCO-NHS (Jena Bioscience, Germany) and subsequently incubated in PBS/Tween for 2 h at room temperature with 2.1 µM of an azide-functionalized oligonucleotide fluorescently labelled with Atto647 (sequence: Atto647N-5′-TCA GTT CAG GAC CCT CGG CT-3′-N3) (custom made by Biomers). Beads were subsequently labelled with FITC (10 mg/mL dissolved in 0.1 M carbonate buffer at pH 9.6) for 30 min at 4 °C, washed, and stirred. The working concentration was 500 µL beads in 500 µL volume. Finally, beads were loaded with C3b-biotin (1 µg/mL) or DNP-PEG-biotin (1 µg/mL) for 60 and 30 min respectively at 4 °C.

For the preparation of IgG1-quenchable beads, the same beads were incubated with 1 μM of a custom made DNP-PEG2-GSGSGSGK(Biotin)-NH2 (Pepscan Therapeutics, The Netherlands), a standard model antigen for antibody studies, and human anti-DNP IgG1 as previously described^74^. For the preparation of C3b + IgG beads increasing concentrations of DNP-biotin were used (0–1–3 ng/mL) combined with 10 nM anti-DNP IgG1.

At the end of the phagocytosis experiment, the fluorescence of oligo-Atto647 beads was quenched by hybridization with 1 µM of the complementary oligonucleotide coupled to BlackBerry Quencher (BBQ) (sequence 5′-AGC CGA GGG TCC TGA ACT GA-3′-BBQ650) (custom made by Biomers) by incubating for 10 min on ice.

### Neutrophil phagocytosis assay

C3b-labelled beads or bacteria (1.5 × 10^7^ particles/mL) and neutrophils were mixed with a ratio of 10:1 in a final volume of 50 µL in a 96-well round-bottom plate. The reaction was incubated for 15 min at 37 °C on a shaking platform to ensure contact. Samples were then fixed for 30 min with 100 µL of cold formaldehyde with a final concentration of 1.5%. (Polysciences, Inc.). Part of the sample was analyzed by flow cytometry (BD FACSVerse, BD Biosciences for beads phagocytosis, and MACSQuant VYB, for bacterial phagocytosis), and the rest was used for imaging. For imaging on a confocal microscope (Leica TCS SP5 II), samples were transferred into 8-well chambers (Sigma-Aldrich) and incubated with 1 µg/mL wheat germ agglutinin Alexa-647 (Thermo Fisher Scientific) and 1 µg/mL 4′,6-diamidino-2-phenylindole (DAPI) (Thermo Fisher Scientific). Cells were allowed to settle on the bottom for 30 min. Samples were imaged using a 63× lens. FITC, mCherry, and Alexa-647 signals were measured using the 488-nm, 561-nm, and 630-nm lasers, respectively, with suited dichroic and emission filter settings.

To block complement receptors before phagocytosis, neutrophils were pre-incubated with 25 μg/mL of anti-CR1, clone 3D9 (gifted by Admar Verschoor)^75^, or anti-CR3, clone ICRF44 (Purified anti-human CD11b, Biolegend, LEAF™) antibodies in phagocytosis buffer. The concentration of the α-CR1 blocking antibody was determined via inhibition of adhesion assay (S7).

For the quenching assay neutrophils were preincubated for 10 min with the blocking antibodies at RT. Subsequently, 20 µL of fluorescent beads (7.5 × 10^7^/mL) were incubated with 60 µL of neutrophils (2.5 × 10^6^/mL) and 20 µL of inhibitor/buffer for 25 min at 37 °C with shaking. Phagocytosis was stopped on ice, samples were split in 2 × 50 µL and mixed with 50 µL buffer or 50 µL quencher (diluted to 2 µM, so final concentration is 1 µM) and put on ice for 10 min. Finally, samples were fixed with PFA and measured by flow cytometry (BD FACSVerse, BD Biosciences). For analysis, both free beads and neutrophils were acquired in the same setting, gated based on FSC/SSC parameters and evaluated for FITC and Atto647 fluorescence signals in the condition of buffer or the presence of the quencher.

### Neutrophil oxidative burst assay

The induction of oxidative bursts of neutrophils was determined as previously described^76^. Briefly, C3b-labelled beads or bacteria were incubated with neutrophils in a luminol solution (150 µM) prepared in HBSS (Lonza) + 0.1% HSA in white microplates. The chemiluminescent signal was then measured continuously for 120 min at 37 °C in a microplate luminometer (Centro XS^3^ LB 960, Berthold). Neutrophils were stimulated with phorbol myristate acetate (PMA) (Sigma-Aldrich) at a final concentration of 2.7 µM as a positive control for ROS production.

### Neutrophil killing assay

The concentration and vitality of C3b-labelled bacteria were checked using SYTOX Blue staining (Thermo Fisher Scientific) and direct bacterial counting via flow cytometry (MACSQuant VYB) to adjust to 2 × 10^8^ colony forming units (CFU)/mL. Next, 10 µL of bacteria were incubated with 90 µL of 1 × 10^7^ neutrophils/mL in sterile siliconized tubes (Sigma-Aldrich) at 37 °C for 45 min on a shaking platform. As a positive control for killing, we used 2% C6-depleted serum. Neutrophils were then lysed with 900 µL of 0.3% saponin in MilliQ water to release intracellular bacteria, and serial dilutions were plated in duplicates on LB-agar plates with ampicillin. Controls with no ampicillin for sterility were always taken. The most representative dilution was considered in the graphs.

### Single-live-cell/single-target phagocytosis experiments

Individual human neutrophils and target particles were selected and manipulated into contact using a dual-micropipette aspiration system mounted on an inverted microscope (Zeiss), as described previously^5,77^. The experiment chamber was filled with HBSS with Ca^2+^ and Mg^2+^ containing 20 mg/mL human serum albumin (MP Biomedicals). All experiments were performed at room temperature.

### Frustrated phagocytosis

Glass coverslips were mounted in a custom chamber assembly. For IgG opsonization, coverslips were incubated with 10 mg/mL bovine serum albumin (BSA) (VWR) and then with 150 μg/mL rabbit polyclonal anti-BSA IgG at room temperature (Thermo Fisher Scientific). For C3b opsonization, coverslips were incubated with 2 mg/mL biotinylated BSA (Thermo Fisher Scientific), then 400 μg/mL neutravidin (Thermo Fisher Scientific) for 30 min at room temperature, and finally with 100 µg/mL C3b-biotin at 4 °C for 15 min. Unless otherwise noted, all incubations were for 1 h at room temperature and all solutions were prepared in PBS. The resultant opsonin density for both C3b and IgG was about 20,000 molecules per square micron (S6).

To validate our surface coating strategy and compare the molecular densities, we labelled our surfaces with fluorescent antibodies and imaged them on a spinning disc confocal microscope. For the IgG surfaces, we labelled with 30 µg/mL anti-human IgG Fc (cross-reacts with rabbit) conjugated to Alexa Fluor-488 (BioLegend), and for C3b surfaces we coated with 10 µg/mL anti-C3b antibody conjugated to FITC. To convert intensity values to actual molecular densities, we utilized the QSC calibration kit (Bangs Laboratories) which captures known amounts of antibody for each calibration bead, allowing us to construct a calibration curve of molecular density vs. intensity for each fluorescent antibody.

In frustrated phagocytosis experiments, isolated human neutrophils were deposited onto select regions of the coated coverslip and imaged using reflection interference contrast microscopy (RICM), a method that allows us to monitor cell-substrate contact with high-resolution. The contact regions over time were segmented by thresholding on intensity and variance using a custom algorithm in MATLAB.

### Data analysis and statistical testing

Flow cytometry data were analyzed in FlowJo X 10.0.7r2, and percentage positive bacteria or cells were based on gating compared to negative controls. Graphpad Prism 8.0 and Origin were used for graph design and statistical analysis, as described in the figure legends.

### Statement of ethics

*UMC Utrecht.* Informed consent was obtained from all subjects in accordance with the Declaration of Helsinki. Approval from the Medical Ethics Committee of the University Medical Center Utrecht was obtained (METC protocol 07-125/C approved on March 1, 2010). *UCDavis*. Written informed consent was obtained from all subjects. The Institutional Review Board of the University of California Davis approved the protocol covering this study.

## Supplementary Information

Supplementary Video 1.

Supplementary Video 2.

Supplementary Figure S1.

Supplementary Figure S2.

Supplementary Figure S3.

Supplementary Figure S4.

Supplementary Figure S5.

Supplementary Figure S6.

Supplementary Figure S7.

Supplementary Legends.

## Data Availability

All data generated or -during this study are included in this published article (and its Supplementary Information files). No datasets were generated or analyzed during the current study.

## Abbreviations

AUC: Area under the curve
BSA: Bovine serum albumin
CFU: Colony forming units
CR: Complement receptor
DAPI: 4′,6-Diamidino-2-phenylindole
DBCO: Dibenzocyclooctynes
FcγR: Fcγ receptor
FITC: Fluorescein isothiocyanate
HBSS: Hank's balanced salt solution
HSA: Human serum albumin
IgG: Immunoglobulin G
ITAM: Immunoreceptor tyrosine-based activation motif
KDO: Keto-deoxyoctulosonate
LPS: Lipopolysaccharide
MFL: Mean fluorescence
NADPH: Nicotinamide adenine dinucleotide phosphate
PBS: Phosphate buffered saline
PEG: Polyethylene glycol
PMA: Phorbol myristate acetate
PMNs: Polymorphonuclear cells
RICM: Reflection interference contrast microscopy
ROS: Reactive oxygen species
SD: Standard deviation
SEM: Standard error of the mean
TED: Thioester domain
