# Supplementary figures and images for: Purified complement C3b triggers phagocytosis and activation of human neutrophils via complement receptor 1
Source: Sci Rep. 2023 Jan 6;13:274. doi: 10.1038/s41598-022-27279-4 (PMC9822988; doi:10.1038/s41598-022-27279-4)

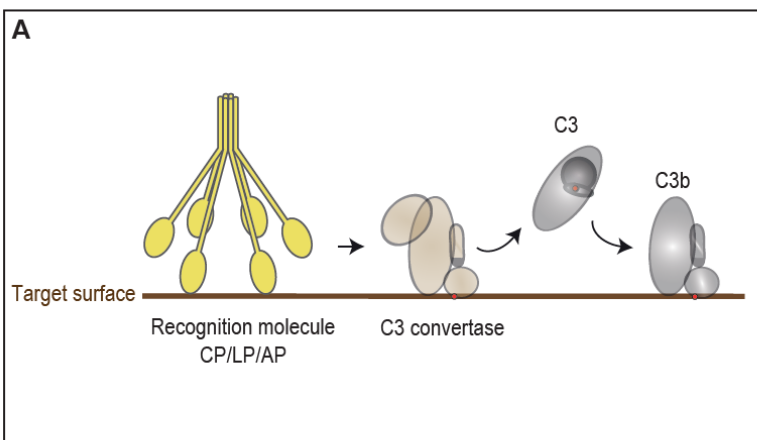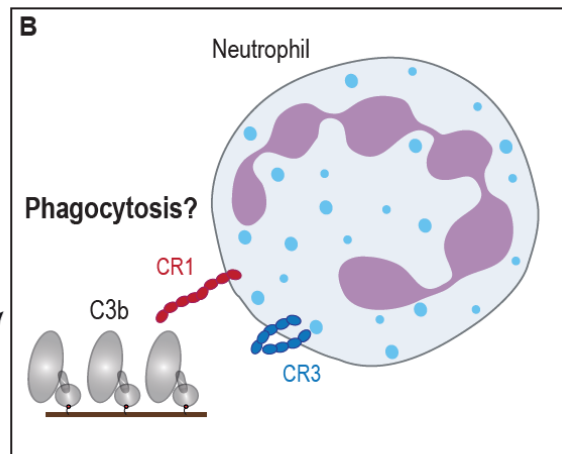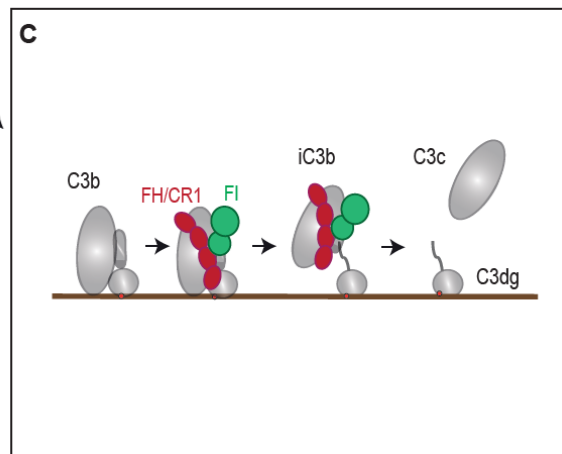

Supplement: Supplementary file 3 — Supplementary Figure S1. [file 41598_2022_27279_MOESM3_ESM.pdf]

S2

A

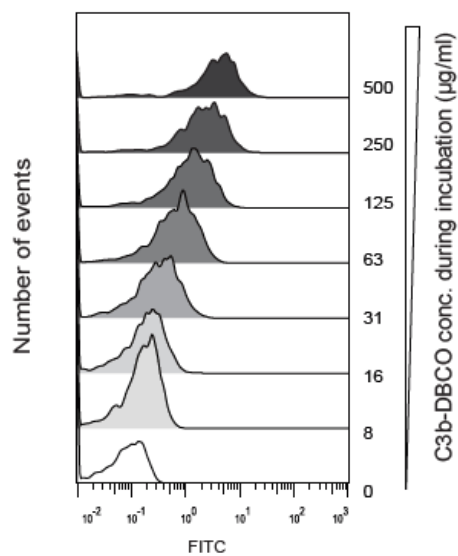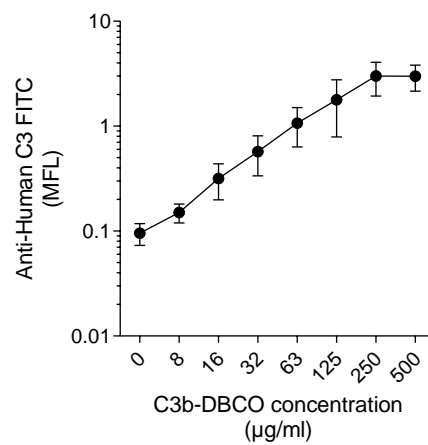

B

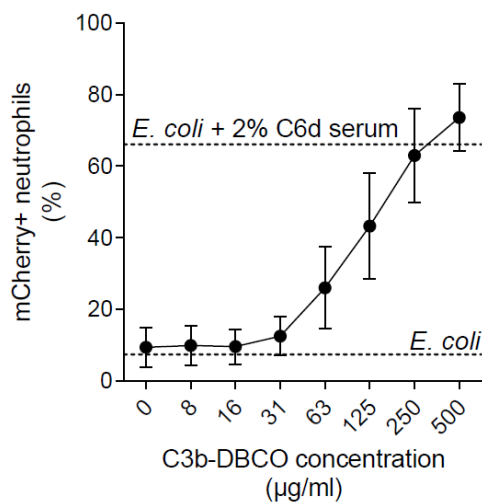

C

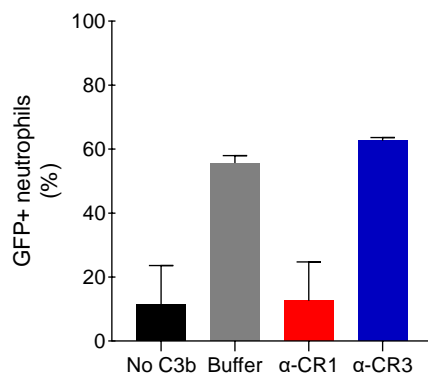

Supplement: Supplementary file 4 — Supplementary Figure S2. [file 41598_2022_27279_MOESM4_ESM.pdf]

A

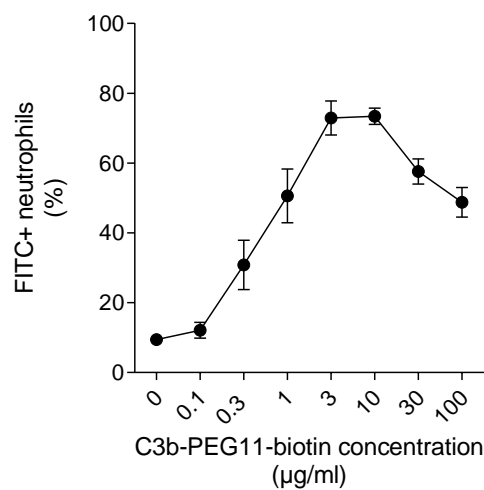

B

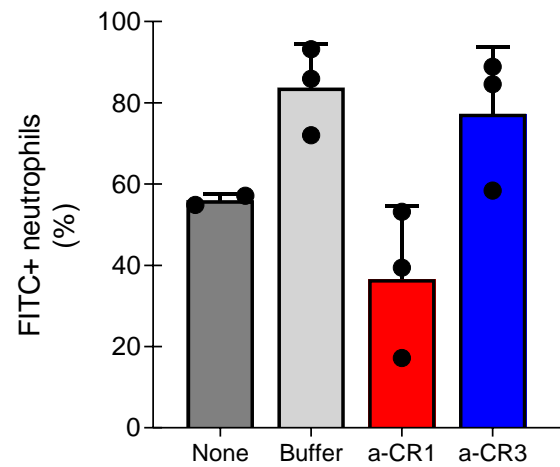

Supplement: Supplementary file 5 — Supplementary Figure S3. [file 41598_2022_27279_MOESM5_ESM.pdf]

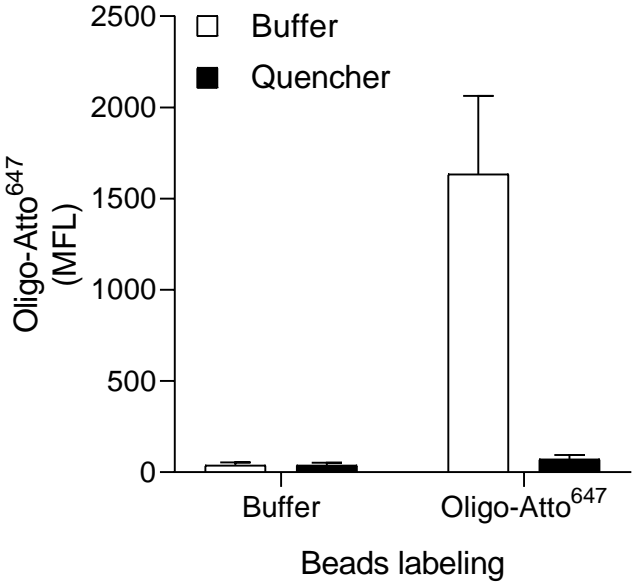

Supplement: Supplementary file 6 — Supplementary Figure S4. [file 41598_2022_27279_MOESM6_ESM.pdf]

A

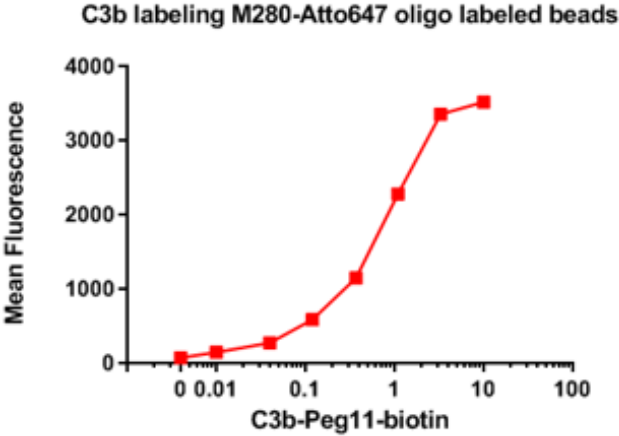

B

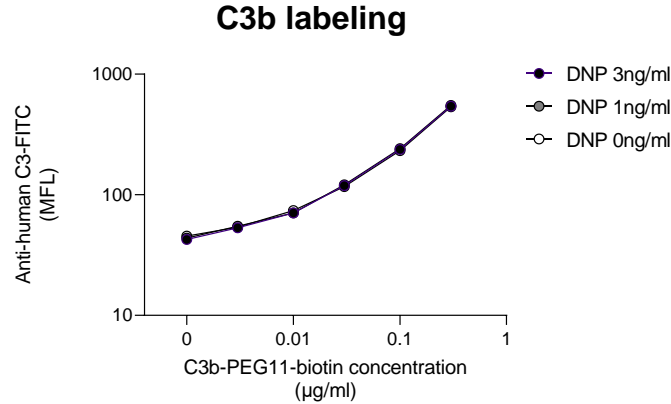

C

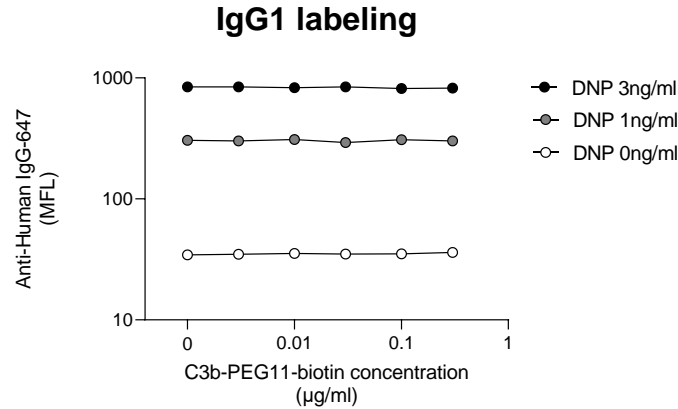

Supplement: Supplementary file 7 — Supplementary Figure S5. [file 41598_2022_27279_MOESM7_ESM.pdf]

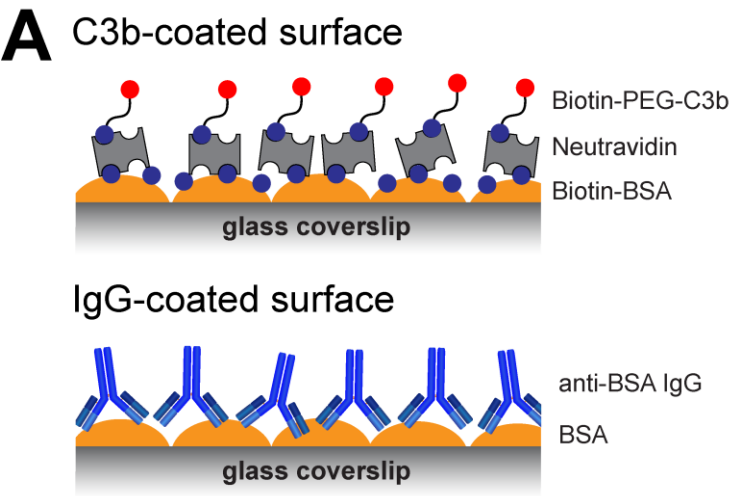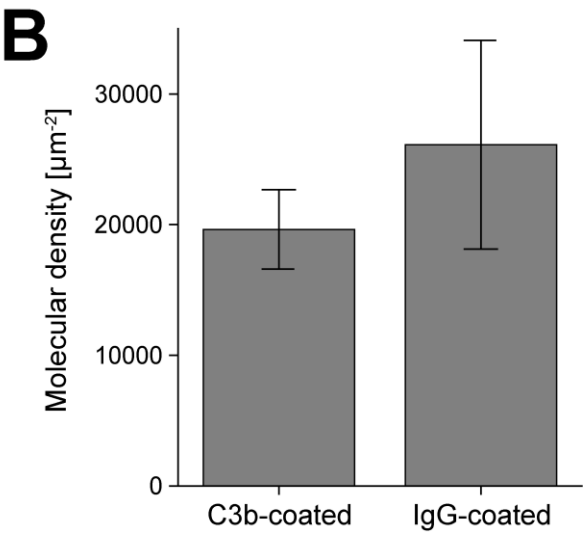

Supplement: Supplementary file 8 — Supplementary Figure S6. [file 41598_2022_27279_MOESM8_ESM.pdf]

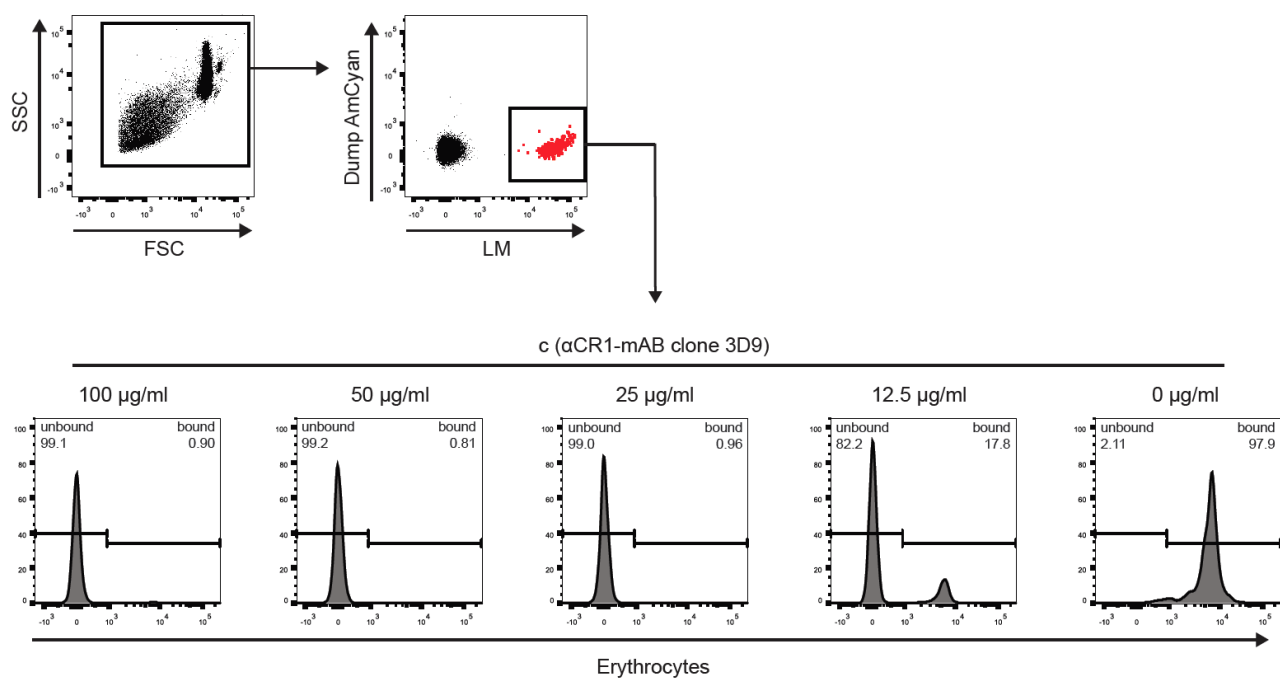

Supplement: Supplementary file 9 — Supplementary Figure S7. [file 41598_2022_27279_MOESM9_ESM.pdf]
