# Supplementary material for: Purified complement C3b triggers phagocytosis and activation of human neutrophils via complement receptor 1
Source: Sci Rep. 2023 Jan 6;13:274. doi: 10.1038/s41598-022-27279-4 (PMC9822988; doi:10.1038/s41598-022-27279-4)
Supplement: Supplementary file 10 — Supplementary Legends. [file 41598_2022_27279_MOESM10_ESM.docx]

### S1. Fate of immobilized C3b on phagocytic targets.

**A)** Upon recognition of foreign cells, all complement activation pathways (classical, lectin and alternative CP/LP/AP) deposit C3 convertases onto the target surface. These enzymes proteolytically cleave soluble C3 to C3b, exposing its reactive thioester moiety (*red*) that covalently attaches onto the surface. **B)** Deposited C3b could induce phagocytosis of targets via interaction with complement receptors 1 or 3 on the surface of neutrophils. **C)** Alternatively, immobilized C3b can be further converted by protease factor I and cofactor factor H or CR1 into “inactive” iC3b and finally C3dg. Immobilized C3b is a ligand for CR1 and CRIg; iC3b is a ligand for CR2, CR3, CR4, and CRIg; C3dg is a ligand for CR2.

### S2. Optimization of C3b-DBCO labelling of *E. coli*

**A)** Representative flow cytometry histograms depicting the fluorescence intensity of Goat anti-human C3-FITC F(ab)”, used to evaluate the deposition of C3b-PEG4-DBCO on mCherry-expressing KDO-N3-labelled MG1655. Histograms are displayed for each concentration of C3b-PEG4-DBCO used during the incubation (*left*). The MFL of Goat anti-Human C3-FITC F(ab)” ± SEM obtained in 3 independent experiments is shown as a function of the C3b-DBCO concentration (*right*). **B)** Percentage of neutrophils engulfing mCherry-expressing C3b-*E. coli* in function of the C3b-DBCO concentration used to label the bacteria. Mean ± SEM of 5 independent experiments. **C)** Percentage of neutrophils engulfing GFP-expressing C3b-*E. coli* upon blocking treatment with no (*gray*), anti-CR1 (*red)* or anti-CR3 (*blue*) blocking antibodies. The percentage of neutrophils interacting with beads not labelled with C3b is shown as negative control (*black*). Mean ± SEM of 3 independent experiments.

### S3. C3b-beads preparation and interaction with human neutrophils.

**A)** Percentage of neutrophils interacting with FITC C3b*-*beads over the total population. Mean ± ± SEM of 3 independent experiments. **B)** Percentage of C3b-beads interacting with neutrophils upon treatment with no (*gray*), anti-CR1 (*red)* and anti-CR3 (*blue*) blocking antibodies. Mean ± SEM of 3 independent experiments.

### S4. Quenching of Atto^647^ beads by oligomer hybridization. Abolition of fluorescence of 2.8-µm streptavidin beads labelled with Oligo-Atto^647^ after treatment with quencher (Oligo-BBQ). MFL ± SD of 7 independent experiments.

### S5. C3b and IgG labeling verification on Oligo-Atto647 beads.

### A) Deposition of C3b-biotin on Oligo-Atto^647^-labelled streptavidin beads is not perturbed by the presence of the fluorophore. B) Deposition of C3b-biotin is not perturbed by simultaneous labeling with DNP-biotin + 10mM anti-DNP IgG1. C) IgG1 deposition on DNP-biotin is not perturbed by labeling with increasing C3b-biotin concentrations.

### S6. IgG and C3b deposition and distribution on glass coverslips for frustrated phagocytosis experiments.

### A) Illustrations of strategies for coating coverslips with C3b or IgG. C3b-coated glass coverslips were prepared by successive incubation with biotinylated BSA, neutravidin, and C3b-biotin. IgG-coated glass coverslips were prepared by first incubating with BSA and then incubating with rabbit polyclonal anti-BSA IgG. B)  Measured surface densities of C3b and IgG on coated surfaces. The mean C3b density was about 20,000 molecules per square micron and the mean IgG density was about 26,000 molecules per square micron. Error bars denote standard deviation.

### S7. Validation of the concentration of anti-CR1 mAb that is needed to achieve blocking. Binding of listeria to erythrocytes in human blood, titration of 3D9 to determine inhibitory concentration. The lowest inhibitory concentration is 25 μg/ml.
